# Structure of a Classical MHC Class I Molecule That Binds “Non-Classical” Ligands

**DOI:** 10.1371/journal.pbio.1000557

**Published:** 2010-12-07

**Authors:** Chee Seng Hee, Song Gao, Bernhard Loll, Marcia M. Miller, Barbara Uchanska-Ziegler, Oliver Daumke, Andreas Ziegler

**Affiliations:** 1 Institut für Immungenetik, Charité-Universitätsmedizin Berlin, Campus Benjamin Franklin, Freie Universität Berlin, Berlin, Germany; 2 Max-Delbrück-Centrum für Molekulare Medizin, Berlin, Germany; 3 Institut für Chemie und Biochemie, Freie Universität Berlin, Berlin, Germany; 4 Institut für Chemie und Biochemie, Abteilung Strukturbiochemie, Freie Universität Berlin, Berlin, Germany; 5 Department of Molecular and Cellular Biology, City of Hope, Beckman Research Institute, Duarte, California, United States of America; 6 Institut für Medizinische Physik und Biophysik, Charité-Universitätsmedizin Berlin, Berlin, Germany; Mount Sinai School of Medicine, United States of America

## Abstract

Chicken *YF1* genes share a close sequence relationship with classical MHC class I loci but map outside of the core MHC region. To obtain insights into their function, we determined the structure of the YF1*7.1/β_2_-microgloblin complex by X-ray crystallography at 1.3 Å resolution. It exhibits the architecture typical of classical MHC class I molecules but possesses a hydrophobic binding groove that contains a non-peptidic ligand. This finding prompted us to reconstitute YF1*7.1 also with various self-lipids. Seven additional YF1*7.1 structures were solved, but only polyethyleneglycol molecules could be modeled into the electron density within the binding groove. However, an assessment of YF1*7.1 by native isoelectric focusing indicated that the molecules were also able to bind nonself-lipids. The ability of YF1*7.1 to interact with hydrophobic ligands is unprecedented among classical MHC class I proteins and might aid the chicken immune system to recognize a diverse ligand repertoire with a minimal number of MHC class I molecules.

## Abstract 2

The chicken MHC YF1*7.1 X-ray structures reveal that this protein binds lipids and thus represents a "hybrid" class I complex with features of classical as well as non-classical MHC molecules.

## Author Summary

Proteins encoded by the major histocompatibility complex (MHC) play crucial roles in vertebrate immune systems, presenting pathogen-derived protein fragments to receptors on effector cells. In contrast, some non-classical MHC class I proteins such as CD1 molecules possess a hydrophobic groove that allows them to display lipids. Chicken *MHC-Y* is a genetic region outside the core *MHC* that harbors several immune-related genes, among them YF1*7.1, which encodes a protein whose structure we solved in this study. YF1*7.1 is an MHC class I molecule that exhibits the architecture typical of classical MHC class I antigens but possesses a hydrophobic binding groove that binds non-peptidic ligands. By using lipid-binding assays, we show that this molecule can indeed bind lipids. Therefore, YF1*7.1 bridges, at least in structural terms, the traditional gap between classical and non-classical MHC class I molecules. Lipid-binding YF1 proteins might serve the chicken to enlarge its otherwise very small repertoire of antigen-presenting MHC class I molecules. Furthermore, comparative analyses of the two protein subunits of classical MHC molecules revealed a structural feature in chickens that appears to be shared by non-mammalian but not by mammalian vertebrates. This unique feature is indicative of a structure-dependent co-evolution of two genetically unlinked genes in non-mammalian species.

## Introduction

Although the immune systems of birds differ in several important aspects from those of mammals, for example in relying on the bursa of Fabricius, and not on bone marrow, for the production of a diverse B cell repertoire [1], the presence of a major histocompatibility complex (MHC) is a unifying feature [2]. The MHC encodes several immunologically relevant proteins, among them classical MHC class I molecules that are membrane-anchored proteins involved in the presentation of foreign or self-protein-derived peptide antigens [3]. Conversely, the products of the evolutionarily distantly related non-classical class I genes (e.g. CD1 loci) can either display non-peptidic ligands such as lipids [4] or bind entire proteins [5]. In the chicken (*Gallus gallus domesticus*), MHC genes are located on the same micro-chromosomal arm in two regions termed *MHC-B* and *MHC-Y* that are physically, but not genetically, linked due to a chromosomal segment that supports a high degree of recombination between the two regions [6].

**Figure 1:**
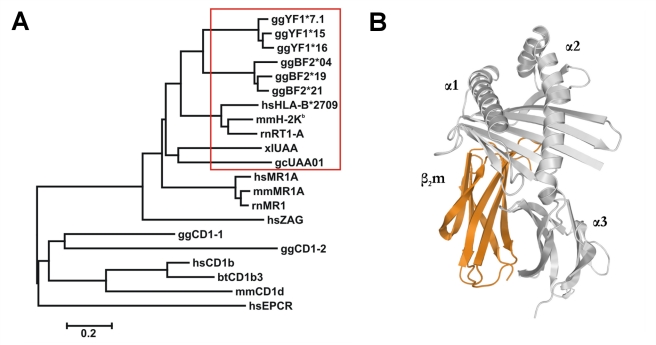
Evolutionary and structural characteristics of YF1*7.1. (A) The evolutionary tree reveals that YF1 isoforms are closely related to chicken MHC-BF2 variants and classical mammalian as well as non-mammalian (frog, nurse shark) class I heavy chains (red box) but are also similar to mammalian MR1 chains and human ZAG. YF1 heavy chains are, however, only distantly related to chicken and mammalian CD1 molecules as well as to EPCR. The designations of the molecules are given in the Accession Numbers section. The tree is drawn to scale, with branch lengths equivalent to evolutionary distances in the units of the number of amino acid substitutions per site. (B) Ribbon diagram of YF1*7.1 non-covalently associated with β_2_m (orange), as seen along the binding groove. A ligand has been omitted for clarity.

The *MHC-B* region resembles the mammalian MHC, e.g. with regard to its influence on the rapid rejection of transplants [7], but has been termed a “minimal essential MHC” due to its small size [8]. It plays a prominent role in genetic resistance, particularly to virally induced tumors [9]. Very near to this region are the only two CD1 genes of the chicken [10],[11]. The *MHC-Y* region, on the other hand, is thought to be associated with a moderate degree of allograft rejection [12] and to influence the fate of tumors induced by Rous Sarcoma virus [13]. It contains at least one polymorphic class I locus, *YF1*, which encodes a class I heavy chain (HC) that associates with β_2_-microglobulin (β_2_m) and is ubiquitously transcribed in both adult and embryonic chickens. The YF1 HC is closely related to that of classical MHC-B and mammalian MHC class I HC but not to non-classical CD1 HC (Figure 1A) [14]. To obtain insights into the role of YF1 molecules in the chicken immune system, we chose a structural approach.

## Results/Discussion

### Basic Structural Features of the YF1*7.1 Molecule

**Figure 2:**
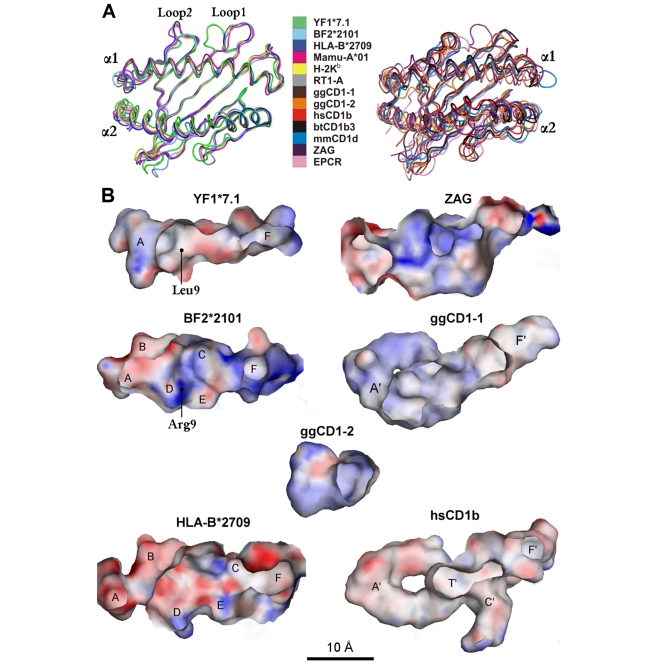
Binding grooves of YF1*7.1 and selected classical or non-classical class I molecules. (A) Overlay of α1- and α2-domains, viewed from above. Classical (YF1*7.1, BF2*2101, HLA-B*2709, Mamu-A*01, H-2K^b^, RT1-A, left) and non-classical class I molecules (all others, right) (see Materials and Methods for details) are superimposed onto the Cα-backbone of the α1-helix and the β-sheet platform, with selected interstrand loops (Loop1 and Loop2) designated. The Loop1 locations of YF1*7.1 and BF2*2101 are nearly indistinguishable but are distinct from those of classical mammalian MHC class I molecules. An interactive three-dimensional (3D) comparison of these molecules is available in Figure S2. (B) Interior molecular surfaces of ligand-devoid binding grooves. The binding pockets of classical (A–F) and non-classical (A', C', F', T') molecules are indicated. The approximate position of HC residues 9 (Leu in YF1*7.1, Arg in BF2*2101) is indicated (see main text for further explanation). Electrostatic potentials are mapped to the molecular surfaces with positive potential (≥20 mV) in blue, neutral potential (0 mV) in white, and negative potential (≤−40 mV) in red. Although the ZAG groove is predicted to bind hydrophobic ligands [24],[31] like CD1 molecules, it appears closely related to that of YF1*7.1 (see also Table S1).

**Figure 3:**
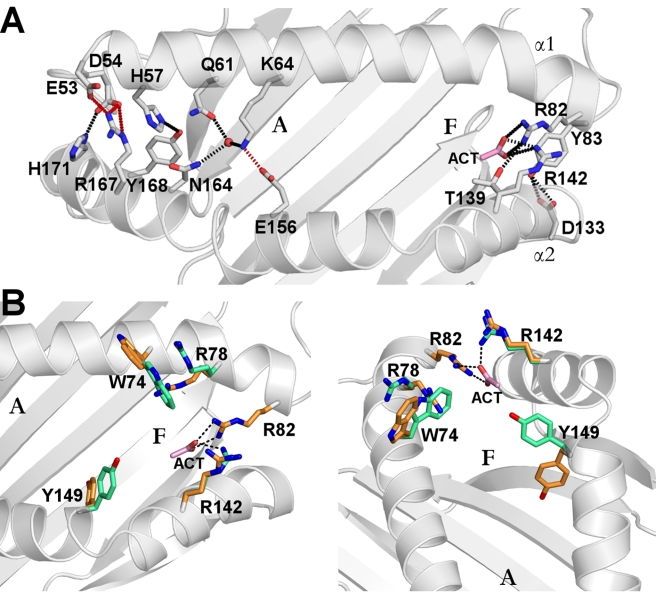
Side chain interactions in the vicinity of the YF1*7.1 binding groove. (A) Side chain interactions between α1- and α2-helices partially cover the A pocket and close the binding groove terminals. Side chains are shown as stick representation with hydrogen bonds and salt bridges indicated by dotted lines. An acetate molecule (ACT) “above” the F pocket is shown as pink stick representation. (B) Two views of the F pocket showing residues that might be involved in ligand binding due to their conformational dynamics. On the left is the view from “above” the binding groove and, on the right, the view from the A pocket along the binding groove towards the F pocket. Residues exhibiting dual conformations are distinguished by orange and green colors. The side chains of Trp74, Arg78, Arg142, and Tyr149 “above” the F pocket have poorly defined electron density compared to the surrounding residues, indicating that they might interact with ligands captured within the F pocket. Also near the F pocket, Arg82 interacts with an acetate molecule, indicating that this HC residue might also be involved in ligand binding.

The complex of YF1*7.1 HC and β_2_m was reconstituted without adding a ligand, and the structure was determined by molecular replacement at 1.32 Å resolution, using the related BF2*2101-β_2_m complex [15] as a search model (Table 1, left column). The YF1*7.1 complex exhibits the typical architecture of classical MHC class I molecules [3], with binding groove-forming, anti-parallel α1- and α2-helices atop of a β-sheet platform. β_2_m and the α3-domain occupy the standard positions below the platform (Figure 1B). However, the YF1*7.1 binding cleft is narrower than that of peptide-presenting MHC class I molecules (Figure 2) and is lined by many hydrophobic residues (16 out of 30 residues forming the binding groove) (Figure S1, Table S1). Charged residues are only found above the floor and at the ends of the groove (Figures 2B, 3A). The volume of the YF1*7.1 binding groove is ∼1,030 Å^3^. This value is considerably smaller than that of typical MHC class I peptide binding grooves (∼1,250–1,900 Å^3^, see e.g. Protein Data Bank entries 1I4F and 1OF2), mammalian CD1 molecules (∼1,800–2,400 Å^3^, 2PO6 and 2H26), or chicken CD1-1 (1,810 Å^3^, 3JVG) (Figure 2B). The YF1*7.1 groove is, however, larger than the miniaturized binding pocket of chicken CD1-2 (∼720 Å^3^, 3DBX) (Figure 2B), which is thought to accommodate maximally a single alkyl chain [16].

**Table 1:** Data collection and refinement statistics.

	YF1*7.1:L1	YF1*7.1:L2
**Data collection**		
Space group	*P*12_1_1	*P*12_1_1
Cell dimensions		
a, b, c (Å)	52.80, 55.47, 63.84	52.87, 55.04, 63.59
α, β, γ (°)	90.00, 96.85, 90.00	90.00, 97.04, 90.00
Resolution (Å)	20–1.32 (1.35–1.32)*	20–1.60 (1.64–1.60)
*R*_merge_ (%)	4.4 (46.1)	4.1 (29.4)
*I*/σ*I*	20.2 (3.0)	17.4 (3.8)
Completeness (%)	96.0 (92.8)	89.8 (93.2)
Redundancy	4.2 (3.6)	2.8 (2.6)
Unique reflections	82,457	43,084
**Refinement**		
*R*_work_/*R*_free_ (%)	15.7/19.0	17.4/21.6
Number of atoms	3,693	3,498
Protein	3,283	3,062
Water	382	386
Ligand	20	38
Other	8	12
B-factors (Å^2^)		
Overall	17.7	17.9
Protein	16.4	16.8
Water	28.1	25.8
Ligand	18.5	26.8
Other	28.6	24.3
R.m.s. deviation		
bond length (Å)	0.013	0.012
bond angle (°)	1.527	1.403

*Value in parentheses represents statistics for data in the highest resolution shell. R.m.s., root mean square.

These comparisons and its hydrophobic character indicated that the YF1*7.1 binding cleft is optimized for the presentation of medium-sized, non-peptidic ligands rather than peptides, despite the overall similarity to classical BF2 molecules of the chicken. This assumption is reinforced by the substitution of Arg9 (in BF2*2101) by Leu9 (in YF1*7.1) on the floor of the binding groove (Figures 2B, S1). Arg9 can assume different conformations that permit a promiscuous anchoring of sequence-unrelated peptides by this dominantly expressed MHC-B class I molecule [15], thus expanding the repertoire of bound peptides. In YF1*7.1, however, the homologous Leu9 residue cannot serve this purpose but contributes instead to the hydrophobic environment of the groove. Another remarkable feature of YF1*7.1 are the bridge-like contacts between several α1- and α2-helical residues that extend over the top of the groove, leaving only its central portion directly accessible to a ligand (Figure 3A). These interactions distinguish YF1*7.1 from BF2*2101 [15] as well as from most [3]–[5] but not all [17] mammalian class I molecules.

Several residues belonging to the end of the α1-helix and the beginning of the α2-helix, i.e. “above” the F pocket of classical MHC class I molecules, are characterized by double conformations (Figure 3B). This suggests the presence of conformational dynamics that may aid in binding structurally distinct ligands to the YF1*7.1 binding groove. The fact that the positive charges of Arg82 and Arg142 are compensated by binding an acetate molecule derived from the crystallization solution (Figure 3) suggests, in addition, that a YF1*7.1 ligand might interact with these two residues.

### Possible Structural Consequences of Allelic Variations

**Figure 4:**
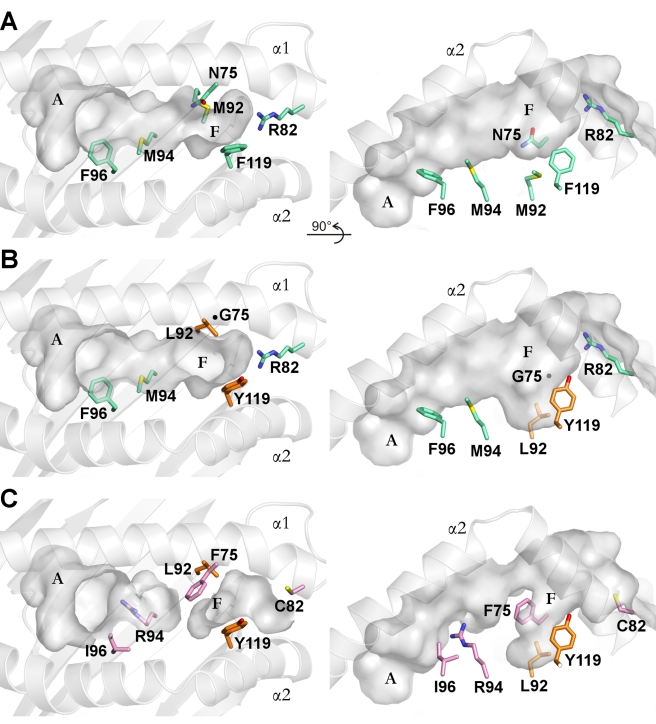
Polymorphic residues of YF1 alleles within the binding groove. Polymorphic residues of YF1*15 and YF1*16 are “mutated” in silico using the YF1*7.1 structure as model. Two views are shown for each allele: on the left, views from “above” the binding groove, and on the right, views “through” the α2-helix. The A and F pockets as well as the α1- and α2-helices are labeled accordingly. (A) Six polymorphic residues that influence the binding groove architectures are shown in green stick representation in YF1*7.1. (B) Substitutions of Asn75Gly and Met92Leu (orange stick representation) in YF1*15 result in a wider groove entrance and deeper F pocket, respectively. The Phe119Tyr exchange slightly narrows the F pocket. The position of Gly75 is shown as a black dot. (C) Six substitutions in YF1*16 result in a division of the binding groove into two parts. The Phe96Ile exchange in YF1*16 slightly enlarges the A pocket, while the substitutions Asn75Phe and Met94Arg are expected to disrupt the middle part of the binding groove. The Asn75Phe exchange also narrows the F pocket together with the Phe119Tyr substitution. The Arg82Cys substitution leaves the F terminal part of the binding groove open, and the Met92Leu substitution results in a deeper F pocket similar to that observed in the case of YF1*15.

Although the sequence of the first 27 amino acids has not been determined for other YF1 alleles (Figure S1), the available information permits us to predict that several exchanges might have an impact on the shape and the electrostatic properties of the binding groove (Figure 4). By modeling the altered residues onto the YF1*7.1 structure, the three exchanges between YF1*7.1 and YF1*15 (Asn75Gly, Met92Leu, and Phe119Tyr) will probably lead to an enlargement of the binding groove (∼1,070 Å^3^ versus ∼1,030 Å^3^), predominantly in the region of the F pocket. In contrast, YF1*16 not only possesses three replacements involving the same residues as in case of YF1*15 but also exhibits three additional exchanges (Arg82Cys, Met94Arg, and Phe96Ile). YF1*16 is thus characterized by pronounced alterations of the groove: the novel Phe at HC position 75 is expected to intersect the cleft, thereby separating the A pocket from the F pocket. This bears some resemblance to amino acid exchanges in mammalian CD1 molecules, where the long T' tunnel of human CD1b is blocked in mouse CD1d molecules due to the replacement of two Gly residues by Leu and Val, respectively [18]. This alteration has consequences for the type of ligands that can be bound by the two CD1 molecules (reviewed in [4]). The volumes of the remaining A (∼490 Å^3^) and F (∼430 Å^3^) pockets of YF1*16 will most likely lead to exposure of the middle section of ligands with two hydrophobic segments, as e.g. in case of phosphatidylcholines (PC) [4]. In addition, the Arg82Cys and the Met94Arg exchanges can be expected to alter the electrostatic properties of the groove. In particular, the novel Arg94 residue at the floor of the binding cleft of YF1*16 might allow the interaction with ligands possessing acidic groups. These allele-specific changes are reminiscent of sequence-dependent alterations that give particular classical MHC class I molecules the opportunity to bind defined sets of ligands [3]. In contrast, the binding grooves of avian and mammalian CD1 molecules do not exhibit such polymorphisms [4],[16],[19]–[21]. These comparisons indicate that, other than in the case of mammalian species, where dissimilar non-polymorphic CD1 *genes* with distinct binding grooves serve to enlarge the repertoire of displayed lipids [4], YF1 *alleles* might be responsible for differential interaction with ligands.

### A Structural Peculiarity Characterizes Chicken Classical Class I Molecules

**Figure 5:**
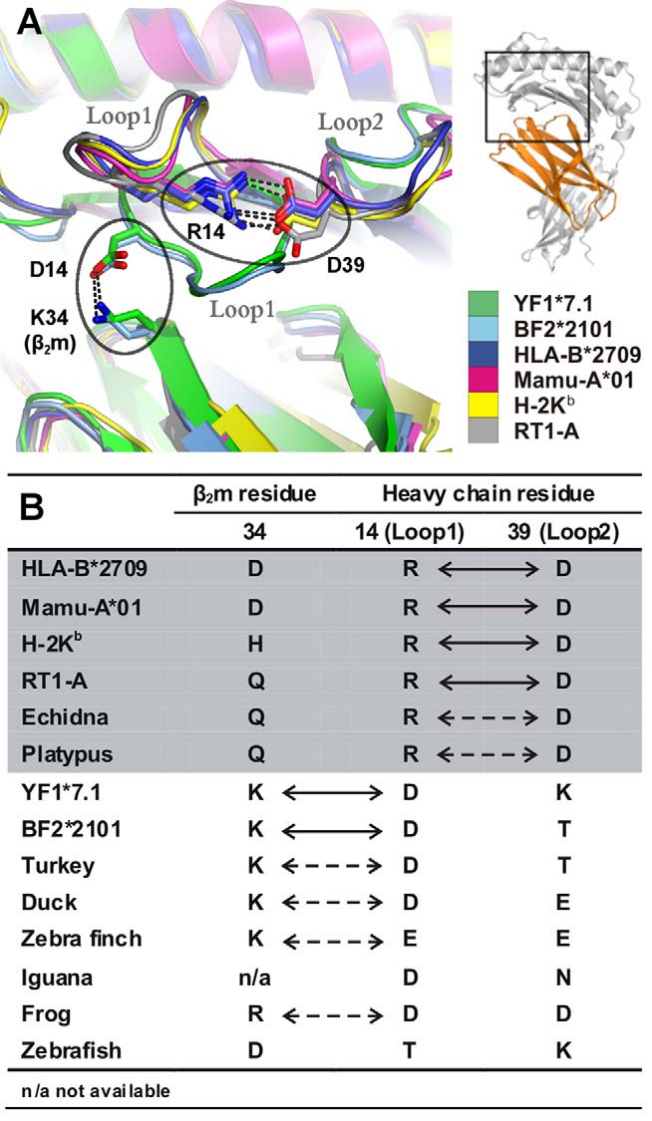
Distinct loop interactions in classical mammalian and chicken MHC class I molecules. (A) Overlay of chicken YF1*7.1 and selected class I molecules reveal that the Loop1 conformations in the YF1*7.1 and BF2*2101 molecules of the chicken deviate from those of mammalian classical class I antigens. This is due to different contacts made by residue 14 of the HC: Asp14 of YF1*7.1 and BF2*2101 interact with Lys34 of β_2_m, whereas Arg14 of HLA-B*2709, Mamu-A*01 (rhesus macaque), H-2K^b^, and RT1-A contact Asp39 of Loop2 (regions of interest indicated by ellipses). Salt bridges are indicated by black dotted lines. The location of the enlarged area within YF1*7.1 is shown on the right, together with a color legend. Carboxyl group atoms of Asp residues and nitrogen side chain atoms of Lys and Arg are colored in red and blue, respectively. (B) Summary of residues involved in the Loop1 - β_2_m or Loop1-Loop2 interactions in various species. Contacts supported by molecular structures are indicated by arrows, and suggested interactions are shown by dotted arrows. The area shaded in grey indicates placental (human, rhesus macaque, mouse, rat) and egg-laying mammals (echidna, platypus).

A further distinct structural feature that YF1*7.1 shares with chicken BF2*2101 molecules [15], but not with classical or non-classical MHC class I molecules from human, rhesus macaque, mouse, rat, cattle, as well as chicken CD1 molecules, is the particular location and conformation of the HC loop 1 (Loop1) (Figures 2A, 5A). This is due to a salt bridge that is formed between Loop1 (residue Asp14 of YF1*7.1 and BF2*2101 molecules) and β_2_m (Lys34). Instead, Arg14 of classical mammalian MHC class I HC contacts Asp39 within HC loop 2 (Loop2) via a salt bridge. Sequence comparisons suggest that the existence of the Loop1-β_2_m contact is probably present also in classical MHC class I molecules from other birds, certain amphibians, and possibly reptiles (Figure 5B). In contrast, the Loop1-Loop2 contact is expected to be restricted to mammals, including egg-laying mammals (monotremes) such as echidna and platypus, indicating that the replacement of the intermolecular contact found in non-mammalian vertebrates by an intramolecular salt bridge is likely to have preceded the development of monotremes, about 170 million years ago [22]. On the other hand, the Loop1-β_2_m interaction probably constitutes an example of structure-dependent co-evolution between two genetically unlinked genes (classical class I HC and β_2_m). Non-classical class I molecules (e.g. CD1 molecules from mammals and chicken, endothelial protein C receptor (EPCR) [23], or Zn-α2-glycoprotein (ZAG) [24]) lack both the Loop1-Loop2 and the Loop1-β_2_m contacts (see also Figures 2A, S1 and interactive Figure S2). This is in line with their evolutionary history, which suggests an early separation of the lineages leading to CD1 and EPCR, on one hand, and to ZAG as well as classical MHC class I molecules, on the other, about 300 million years ago (Figure 1A) [16],[19],[25]. Although no structure has yet been determined for mammalian MHC class I-related (MR)1 molecules [26], their predicted Loop1 and Loop2 do not appear to be connected (Asp14, Val39; Figure S1). Likewise, a salt bridge-mediated interaction between Loop1 and mammalian β_2_m (Asp, His or Asn34; Figure 5B) is also not obvious.

### Thermodynamic Behavior of the YF1*7.1 Complex

The thermodynamic properties of MHC class I molecules are crucially influenced by the presence of ligands within the binding groove [27]. Therefore, we sought to gain insight into the stability of the YF1*7.1 complex using differential scanning calorimetry (DSC). As a consequence of a higher degree of inter-experimental variability than in case of peptide-binding MHC class I complexes (e.g. HLA-B27 molecules) [28], the thermodynamic behavior of the complex could not be determined reliably. We observed, however, that the “melting” temperature of the YF1*7.1 complex was considerably decreased in comparison with typical peptide-presenting mammalian MHC class I molecules (Figure S3), since its dissociation began already at ∼40°C. This value is lower than the body temperature of a chicken (∼41.8°C) [29], indicating that the YF1*7.1 complex exhibits only a limited degree of structural integrity and might thus be prone to interaction with a ligand that could confer an improved stability in vivo.

### Non-Peptidic Ligands in the YF1*7.1 Binding Groove

**Figure 6:**
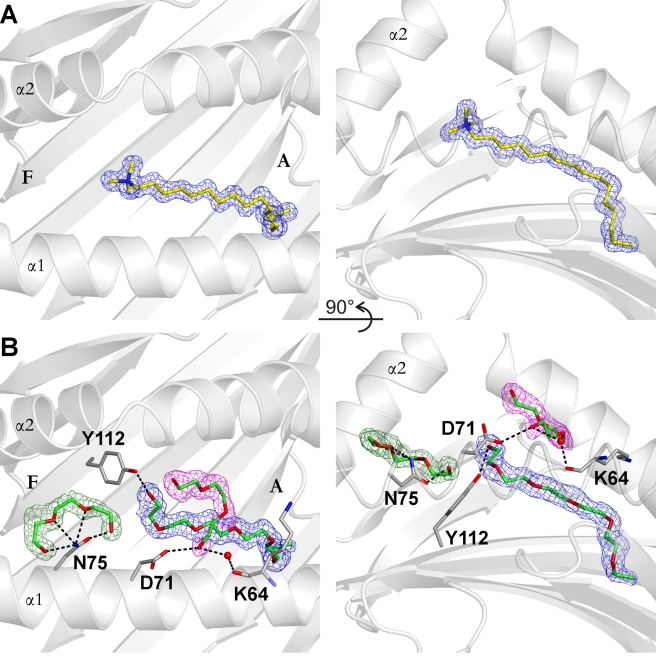
Electron densities observed in YF1*7.1 structures. The electron densities derived from 2*F*o–*F*c maps after refinement are shown as blue, magenta, and green meshes with a contour level of 1σ. Two different types of electron densities (resembling those depicted in A or B) can be observed in eight data sets collected under different cryo-conditions. Side chains interacting with ligands are shown as grey stick representation, with oxygen and nitrogen atoms indicated with red and blue color, respectively. Hydrogen bonds are shown as black dashed lines. Two views are displayed for each type of electron density: on the left, views from “above” the binding groove, and on the right, views from the α1-helix (visible as loop in the foreground). (A) Electron density of the YF1*7.1 complex without an added ligand cryo-protected with glycerol (YF1*7.1:L1). (B) Electron density of the YF1*7.1:L2 complex, using PEG 200 for cryoprotection. Three α1-helical residues are involved in indirect (Lys64) or direct (Asp71, Asn75) contacts to the PEG 200 molecules.

Due to the high-resolution density map of the YF1*7.1 structure, we were also able to model a ligand (L1) with a linear chain of 17 atoms and a tetragonal head group in the groove (Figure 6A). This ligand is buried within the binding groove, with the end of its tail (∼6 backbone atoms) inserted deeply into the hydrophobic A pocket and a tetragonal head group located in the middle of the groove entrance (Figure 6A). The binding of this ligand resembles the situation observed for CD1 molecules of the chicken, bovine, mouse, and human, where unidentified hydrophobic molecules, some of considerable length, have also been found (see e.g. interactive Figure S2, “UL–ggCD1-1” [19], “palmitate–ggCD1-2” [16], and “spacer-hsCD1b” [20]). These unidentified ligands are thought to stabilize CD1 molecules and may facilitate the interchange with other ligands both inside and outside of the cell [4],[19],[20]. Although we suspected the YF1*7.1 ligand to be cetrimonium (hexadecyltrimethylammonium), an antiseptic cationic surfactant, various approaches including mass spectrometric analyses did not allow us to identify L1 unambiguously.

Nevertheless, this detection of a hydrophobic molecule bound to YF1*7.1 as well as the hydrophobic character of the narrow binding groove suggest that the natural ligands for this protein might be lipids and not peptides. We therefore attempted to form complexes with lipids that are typical cellular components and that YF1*7.1 might encounter within the endoplasmic reticulum during or after assembly. These included palmitoyloleoylphosphatidylcholine (POPC), dioleoylphosphatidylcholine (DOPC), palmitic acid (PLM), oleic acid (OLA), and PC. Following reconstitution and chromatographic analyses, we obtained several YF1*7.1 complexes for crystallization trials. Polyethyleneglycol (PEG) 4000 was always necessary to obtain crystals, but we used either glycerol or PEG 200 as cryoprotectants. For nearly all complexes, diffraction data sets at resolutions of 1.5–1.7 Å were obtained (Table S2). The refined structures reveal that reconstitution of YF1*7.1 in the presence of these lipids does not result in additional electron density within the binding groove that can unequivocally be assigned to any of these ligands, suggesting that the examined lipids are not bound by this protein.

In contrast, the use of PEG 200 for cryoprotection (Tables 1, S2) invariably leads to the presence of two additional stretches of electron density within the binding groove, which can best be modeled as two PEG 200 molecules, in addition to a longer fragment exhibiting similarity to the unidentified ligand of YF1*7.1:L1 (YF1*7.1:L2; Figure 6B). In the absence of another plausible explanation, we modeled a fragment of PEG 4000 into this electron density, with the hydrophobic terminus extending into the depth of the A pocket. The identification of the two short ligands as PEG 200 is supported by the characteristic horseshoe-like shape [30],[31], which is particularly obvious in case of the molecule within the F pocket. The conformation of this ligand is maintained by multiple predicted contacts to Asn75. In turn, these lead to a reorientation of the larger ligand, which can now be contacted directly by Tyr112. The second small ligand is located above the larger molecule but is still buried completely within the YF1*7.1 complex. No detectable electron density towards the F pocket is present in the YF1*7.1:L1 structure, suggesting that disordered water molecules occupy this section of the binding groove. The bound hydrophobic chains indicate that natural ligand(s) might occupy comparable positions.

A schematic representation of the types of natural ligands that could possibly bind to the YF1*7.1 groove is provided in Figure S4. A ligand with a long, hydrophobic tail would fit ideally into the highly hydrophobic environment of the A pocket (Figure S4A). Further hydrophobic ligands as seen in the YF1*7.1:L2 structure might bind in addition, e.g. to the F pocket (Figure S4B). However, this would leave Arg82 and Arg142 without charge compensation. Our detection of an acetate molecule that is bound to these residues provides evidence that such salt bridge-mediated interactions could be favored (Figure S4A,B), indicating that a negatively charged head group of a lipid might be accommodated in or “above” the F pocket (Figure S4C). Comparable charge compensatory interactions are occasionally observed between negatively charged lipidic head groups and positively charged membrane protein residues such as arginine and lysine [32],[33], and contacts involving the side chain of α1-helical arginine residues (Arg73, Arg79) can also be found in case of CD1:ligand interactions [21],[34]–[36]. We consider it unlikely that lipids would bind with hydrophobic portions into the F pocket, unless a negatively charged head group of a lipid would be positioned above the groove and permit Arg78, Arg82, or Arg142 to form contacts with the ligand (Figure S4D). The conformational flexibility of arginine residues might facilitate such interactions.

The existing ambiguities in modeling ligands into the observed electron density and in identifying them are likely to be also a reflection of considerable dynamics exhibited by YF1*7.1-bound molecules. Wang and co-workers detected a comparable phenomenon in the case of two lipids bound to mouse CD1d molecules [21]. The difficulties in assigning ligands for YF1*7.1 molecules may be compared to those encountered by Bjorkman and co-workers with the human ZAG protein [31]. Despite extensive attempts and the prediction that the ZAG binding groove might accommodate hydrophobic molecules (compare Table S1), no natural ligand for the binding cleft of this protein has so far been identified. Although ZAG and YF1*7.1 are evolutionarily related (Figure 1A), there are also pronounced differences between them: ZAG lacks an association with β_2_m and instead interacts with prolactin-inducible protein [37], possesses a larger binding groove (Figure 2B), is non-polymorphic, and exists as a secreted molecule [24],[31]. The closest mammalian relatives of YF1*7.1 molecules could be the MR1 antigens of human, mouse, and rat (Figure 1A). Although there is evidence that these molecules might bind lipids and are recognized by a specialized subpopulation of T cells [26],[38],[39], it is currently difficult to judge to what extent these comparisons are valid, since structures for MR1 antigens have so far not been reported.

### Ligand Search for YF1*7.1 Molecules

**Figure 7:**
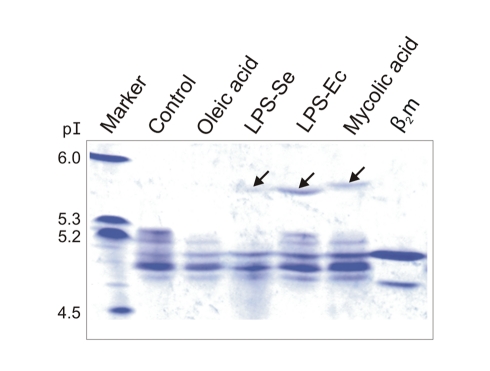
Isoelectric focusing analysis of YF1*7.1 complexes incubated with lipid preparations. The pI of marker protein (lane left) is indicated. The analysis comprised (I) YF1*7.1 without added lipid, (II) YF1*7.1 with oleic acid, (III) YF1*7.1 with lipopolysaccharide from *S. enterica*, (IV) YF1*7.1 with lipopolysaccharide from *E. coli*, (V) YF1*7.1 with mycolic acid, and (VI) monomeric β_2_m. The arrows indicate the positions of novel bands obtained following incubation of YF1*7.1 complexes with selected lipid preparations.

Since our structural studies did not yield support for an endogenous ligand within the YF1*7.1 binding groove, we attempted to bind also a number of nonself lipids to this molecule, employing native isoelectric focusing (IEF). Instead of reconstituting YF1*7.1 with various lipid preparations, we incubated these potential ligands with the reconstituted, purified HC:β_2_m complex and performed IEF (Figure 7). The unloaded YF1*7.1 complex migrates as one major species and four minor components of which one appears to be due to free β_2_m. Incubation with oleic acid did not alter this pattern, but incubation with a mixture of lipopolysaccharides from *Salmonella enterica* or *Escherichia coli* revealed a novel band with a pI of ∼5.6. In addition, a mycolic acid preparation from *Mycobacterium tuberculosis* yielded a novel species with a pI of ∼5.7. These results indicate that in some of the YF1*7.1 molecules, a charged species has replaced the molecule(s) that may have been loaded into the binding groove. Similarly, as in the case of CD1 molecules, charged lipids do not always bind to all proteins within the preparation, leading to a pI shift only in a subpopulation of the molecules [20],[40].

## Conclusions

The results presented here attest to the unusual versatility of classical MHC class I antigens. Not only do they display peptides [3],[15],[28] or post-translationally modified protein fragments [41],[42], but they may also bind hydrophobic ligands, as shown here. The prerequisites for the latter characteristic are so far met only by the YF1*7.1 molecule, which can be regarded as a “hybrid” structure, favoring the interaction with “non-classical” ligands through the combination of a hydrophobic binding groove with a classical scaffold. As there is only a single fully functional, polymorphic classical MHC class I gene, BF2, in most MHC-B haplotypes [43],[44], and just one non-polymorphic CD1 molecule is available to display complex lipids (CD1-1) [19], the chicken's antigen-presenting capabilities might be limited. Together with the unusual, massive expansion of highly polymorphic immunoglobulin-like loci (“CHIR”) within the leukocyte receptor complex [45],[46], lipid-binding YF1 molecules might be part of a strategy to overcome inadequacies in the repertoire of displayed ligands and thus improve the ability of this species to fight successfully against infections [15],[16],[19],[44],[45].

The analysis of MHC-encoded class I molecules of the chicken lags far behind that of mammals, so that many questions, e.g. with regard to the nature of the cellular interaction partners of YF1 and CD1 molecules, are currently unsolved. However, our analyses demonstrate that YF1 molecules deserve to be studied in more detail, because they bridge, at least in structural terms, the traditional gap between peptide-presenting classical and lipid-displaying non-classical class I molecules.

## Materials and Methods

### Protein Purification and Crystallization

Procedures for protein preparation and crystallization of YF1*7.1 HC and β_2_m have been reported previously [47]. A similar procedure was applied to produce the other YF1*7.1 complexes. In the reconstitution experiments, however, the respective lipid was added in 10-fold molar excess to YF1*7.1 HC in a buffer pre-warmed to 37°C, followed by incubation at room temperature for 2 d. All lipids were obtained from Sigma-Aldrich with the following product numbers: palmitic acid (PLM), P5585; oleic acid (OLA), O1008; phosphatidylcholine (PC), P2772; dioleoylphosphatidylcholine (DOPC), P6354; and palmitoyloleoylphosphatidylcholine (POPC), P3017. Lipids were dissolved in dimethylsulfoxide (DMSO) and the concentration adjusted to 10 mg/ml and preheated to ∼40°C prior to adding them to the reconstitution experiments of YF1*7.1 HC and β_2_m. Protein complexes were purified and crystallization experiments were performed as described before [47]. Crystals were cryo-protected with the respective reservoir solution supplemented with either 19% (v/v) glycerol or 19% (v/v) PEG 200.

### Data Collection and Structure Determination

X-ray diffraction data sets were collected at BESSY II, Berlin, Germany, at beamline 14.1 or 14.2. Structure solution of the YF1*7.1 complex has been described by us [47]. Molecular replacement for the other structures was performed by employing a search model of ligand- and water-depleted YF1*7.1:L1. Crystals of the YF1*7.1:L2 complex were isomorphous to the initially determined YF1*7.1:L1 complex. Restrained and TLS refinement with 3 TLS groups designated for α1-α2 domain, α3 domain, and β_2_m were then carried out with Refmac5 [48] and the model building was performed with COOT [49]. The refined models show excellent steric and geometric quality and have no residue in the disallowed region of the Ramachandran plot, as assessed with MolProbity [50].

### Structure Presentation and Computational Analyses

Figures depicting structures were prepared with PyMOL [51]. In silico mutagenesis for YF1*15 and YF1*16 alleles (Figure 4) was performed using PyMOL. The polymorphic residues were substituted in the YF1*7.1 structure and the side chain conformations were chosen based on the frequencies calculated by the program, taking into consideration that the side chains do not clash and have least van der Waals overlay with the surrounding residues. Electrostatics potentials of binding grooves in Figure 2B were calculated with ABPS tools [52] embedded in PyMOL. Binding groove volumes were calculated using the web-based program CASTp with a probe radius of 1.4 Å [53]. The procedure to create interactively accessible 3D images and to integrate them into a PDF document using Adobe Acrobat 9 Pro Extended as well as Adobe 3D Reviewer (as in the interactive Figure S2) has been described [54]. For viewing, the latest version of the freely available Adobe Reader 9 should be installed. The sequence alignments (Figure S1) were generated with Clustal W [55]. Evolutionary analyses (Figure 1A) were conducted using the Neighbor-Joining method [56] in program MEGA4 [57]. The evolutionary distances were computed using the Poisson correction method [58]. All positions containing alignment gaps and missing data were eliminated only in pairwise sequence comparisons (Pairwise deletion option).

### Differential Scanning Calorimetry (DSC)

For DSC, the YF1*7.1:L1 complex and the separately reconstituted β_2_m samples were prepared in a buffer containing 10 mM phosphate (pH 7.5) and 150 mM NaCl at a protein concentration of 0.2 mg/ml as determined by UV absorption at 280 nm. Molecular masses and extinction coefficients were calculated from the amino acid composition using the ProtParam tool on the ExPASy-server (www.expasy.ch/tools/protparam.html). DSC measurements and the determination of melting temperature (*T*_m_) values were carried out as previously described [59]. The data were analyzed using the “ORIGIN for DSC” software package.

### Isoelectric Focusing (IEF)

YF1*7.1 complexes and β_2_m used for IEF were purified as described previously [47]. Briefly, the protein complexes in a buffer composed of 20 mM Tris pH 7.5 and 150 mM NaCl were incubated at 37°C for 2 d with the respective lipid dissolved in DMSO at a 1∶10 (YF1*7.1:lipid) molar ratio. Lipids were purchased from Sigma-Aldrich with the following product numbers: oleic acid (O1008), lipopolysaccharides from *Salmonella enterica* (L2525), lipopolysaccharides from *Escherichia coli* (L5293), and mycolic acid from *Mycobacterium tuberculosis* (M4537). Five µg of protein sample and 10 µg IEF Marker 3–10 (Invitrogen) were applied to the native IEF gel at a pH range of 3–7 (Invitrogen). Electrophoresis was performed at 4°C using the XCell SureLock Mini-Cell system (Invitrogen) according to manufacturer's protocol. Proteins were detected by staining with Coomassie Brilliant Blue R 250 (SERVA).

### Accession Numbers

The National Center for Biotechnology Information (NCBI) accession numbers (http://www.ncbi.nlm.nih.gov) for proteins discussed in this article are as follows: chicken YF1*7.1 (AF218783), YF1*15 (AY257165), and YF1*16 (AY257166); chicken BF2*04 (Z54323), BF2*19 (Z54360), and BF2*21 (AF013493); human HLA-B*2709 (Z33453); mouse H-2K^b^ (P01901); rat RT1-A (M31018); frog UAA (L20733); nurse shark UAA01 (AF220063); human MR1A (AJ249778); mouse MR1A (AF010448); rat MR1 (Y13972); human ZAG (M76707); chicken CD1-1 (AY874074) and CD1-2 (AY375530); human CD1b (AL121986); bovine CD1b3 (Q1L1H6); mouse CD1d (AK002582); and human EPCR (AF106202).

The Research Collaboratory for Structural Bioinformatics (RCSB) Protein Data Bank accession numbers (http://www.pdb.org) for the YF1*7.1:L1 and YF1*7.1:L2 structures are 3P73 and 3P77, respectively. The accession numbers for other proteins discussed in this article are as follows: BF2*2101 (3BEV), HLA-B*2709 (1OF2), Mamu-A*01 (1ZVS), H-2K^b^ (1S7Q), RT1-A (1KJM), ZAG (1ZAG), ggCD1-1 (3JVG), ggCD1-2 (3DBX), hsCD1b (2H26), btCD1b3 (3L9R), mmCD1d (2FIK), and EPCR (1LQV).

## Supporting Information

Figure S1 **Amino acid sequence alignment of the α1- and α2-domains of YF1 alleles with selected classical and non-classical MHC class I molecules.** Numbering refers to YF1*7.1. Secondary structure is shown at the top of the alignment: known or predicted α-helices as pink bars and β-sheets as blue bars. Residues with side chains contributing to the binding grooves (crystallographic evidence) are colored according to their biochemical properties: acidic as red, basic as blue, polar as green, and hydrophobic as yellow. Residues contributing to pockets A and F of classical class I molecules are marked with “A” and “F” above the alignment, respectively. The sequences of YF1*15 and YF1*16 are only partial and lack the first 27 amino acids (indicated by dots).
(1.15 MB EPS)

Figure S2 Binding grooves of YF1*7.1 and selected classical or non-classical class I molecules, together with an embedded interactive three-dimensional figure. The three-dimensional (3D) comparison of the molecules in (A) can be activated by clicking on the image in (B). Each individual structural component (with its designation shown on the left panel) can be selected or removed by checking the boxes in the model tree, using the mouse buttons. A tree of all available models is available through clicking onto the respective icons to the right of the “Views” drop-down menu. Each model can be manipulated individually (the tools to rotate, pan, or zoom can be selected through the toolbar or the contextual menu). Preset views (shown below the model tree) can be selected in the form of a “tour” by clicking the green arrows in the middle of the opened model tree menu. Termination of the interactive session can be accomplished by right-clicking anywhere onto the model and choosing “Disable 3D.”
(2.22 MB PDF)

Figure S3 Thermodynamic stabilities of YF1*7.1 complexes and β_2_m measured by differential scanning calorimetry. Examples for experimental excessive heat capacity curves (black curved lines) and deconvolution results (red curved lines) of (A) a YF1*7.1:L1 complex and (B) free β_2_m. The experimental curve of the YF1*7.1:L1 complex can be deconvoluted into three two-state transitions with *T*_m_^1^ = 47.7°C, *T*_m_^2^ = 57.9°C, and *T*_m_^3^ = 64.1°C, while only one two-state transition can be deconvoluted for β_2_m (*T*_m_ = 59.1°C).
(0.27 MB TIF)

Figure S4 Schematic representation of YF1*7.1 ligand binding modes. The area around the A pocket is marked in green, that around the F pocket in blue; the lengths of the ligands are approximations. (A) The binding of a hydrophobic ligand with a long aliphatic chain within the A pocket is depicted (compare Figure 6A); an acetate molecule forms salt bridges with Arg82 and Arg142. (B) A hydrophobic ligand within the A pocket is shown, together with two short aliphatic molecules (compare Figure 6B); an acetate molecule compensates the charges of Arg82 and Arg142. (C) A large hydrophobic ligand with a negatively charged head group occupies most of the binding groove and interacts with the positively charged amino acids in the vicinity of the F pocket. (D) A ligand with branched hydrophobic segments rests within the binding groove; its exposed head group might interact with positively charged residues at the surface of the YF1*7.1 complex.
(1.07 MB TIF)

Table S1 Comparison of binding groove residues of YF1*7.1 as well as selected classical and non-classical class I molecules.
(0.03 MB DOC)

Table S2 **Crystallization and cryo-protectant conditions.**
(0.03 MB DOC)

## Abbreviations

3D: three-dimensional
β_2_m: β_2_-microglobulin
DOPC: dioleoylphosphatidylcholine
DSC: differential scanning calorimetry
HC: heavy chain
IEF: isoelectric focusing
L1 and L2: unidentified ligands
MHC: major histocompatibility complex
OLA: oleic acid
PC: phosphatidylcholine
PEG: polyethyleneglycol
PLM: palmitic acid
POPC: palmitoyloleoylphosphatidylcholine
