# Supplementary figures and images for: Structure of a Classical MHC Class I Molecule That Binds “Non-Classical” Ligands
Source: PLoS Biol. 2010 Dec 7;8(12):e1000557. doi: 10.1371/journal.pbio.1000557 (PMC2998441; doi:10.1371/journal.pbio.1000557)

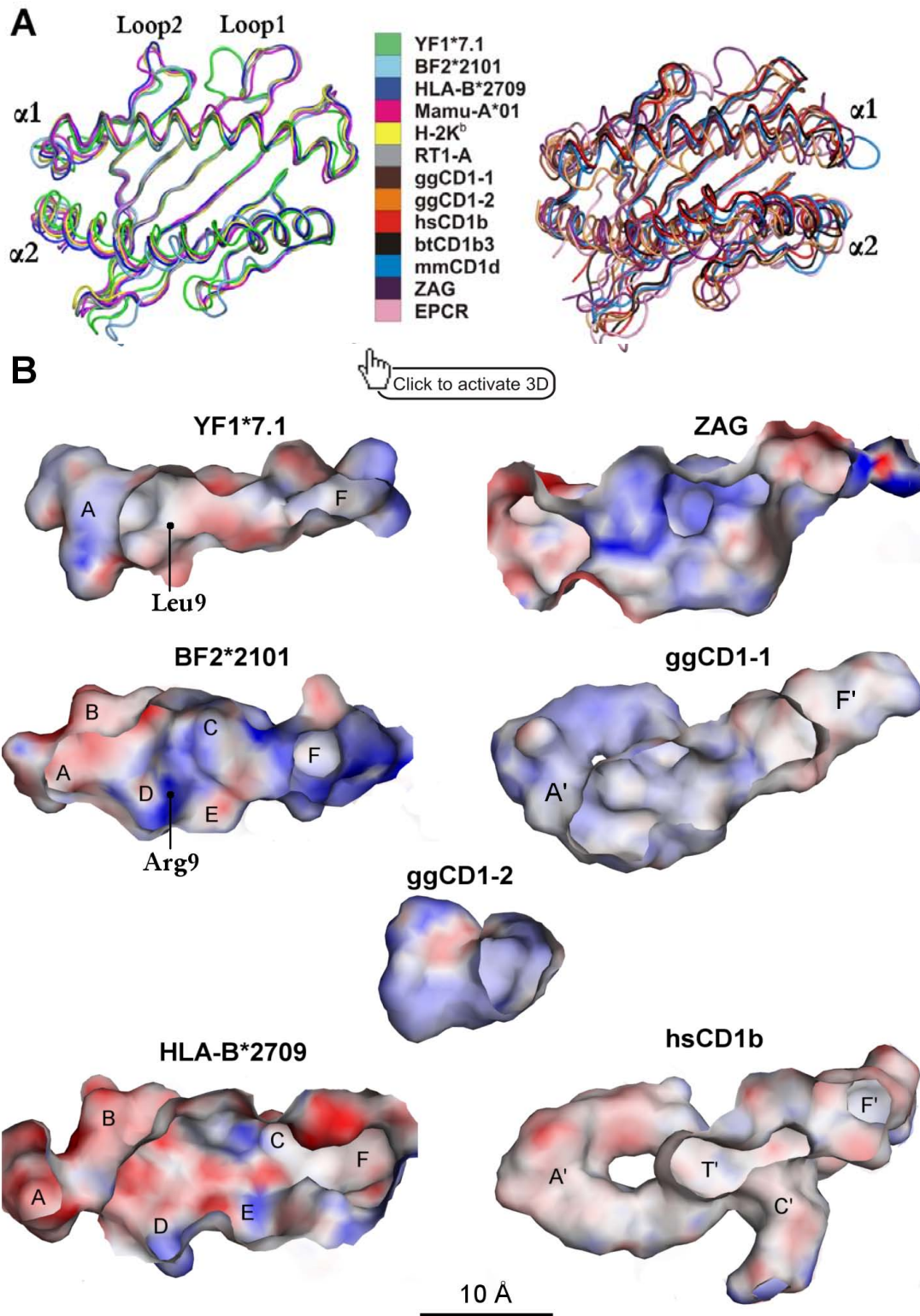

Supplement: Figure S2 — Binding grooves of YF1*7.1 and selected classical or non-classical class I molecules, together with an embedded interactive three-dimensional figure. The three-dimensional (3D) comparison of the molecules in (A) can be activated by clicking on the image in (B). Each individual structural component (with its designation shown on the left panel) can be selected or removed by checking the boxes in the model tree, using the mouse buttons. A tree of all available models is available through clicking onto the respective icons to the right of the “Views” drop-down menu. Each model can be manipulated individually (the tools to rotate, pan, or zoom can be selected through the toolbar or the contextual menu). Preset views (shown below the model tree) can be selected in the form of a “tour” by clicking the green arrows in the middle of the opened model tree menu. Termination of the interactive session can be accomplished by right-clicking anywhere onto the model and choosing “Disable 3D.” (2.22 MB PDF) [file pbio.1000557.s002.pdf]

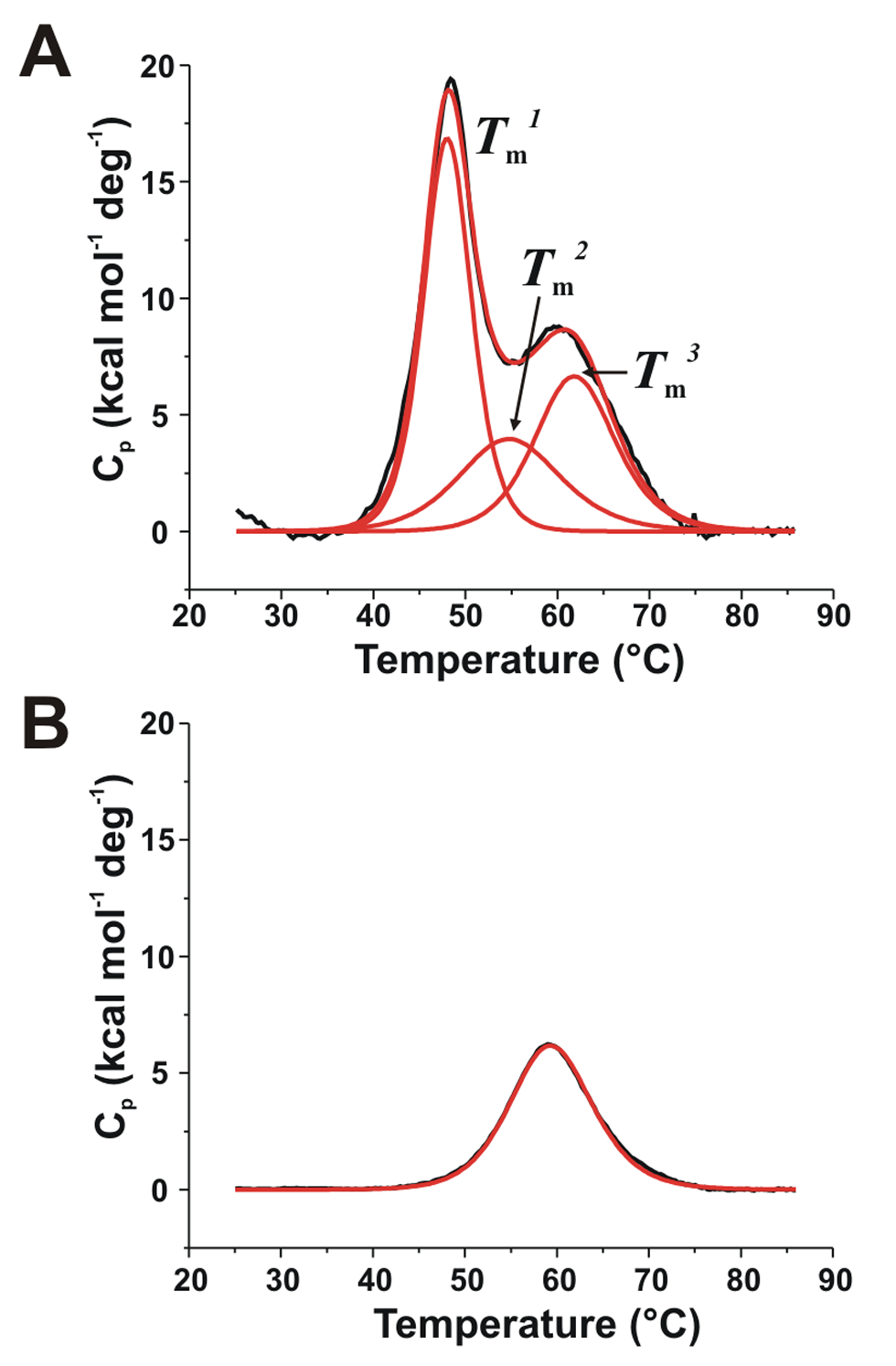

Supplement: Figure S3 — Thermodynamic stabilities of YF1*7.1 complexes and β2m measured by differential scanning calorimetry. Examples for experimental excessive heat capacity curves (black curved lines) and deconvolution results (red curved lines) of (A) a YF1*7.1:L1 complex and (B) free β2m. The experimental curve of the YF1*7.1:L1 complex can be deconvoluted into three two-state transitions with T m 1 = 47.7°C, T m 2 = 57.9°C, and T m 3 = 64.1°C, while only one two-state transition can be deconvoluted for β2m (T m = 59.1°C). (0.27 MB TIF) [file pbio.1000557.s003.tif]

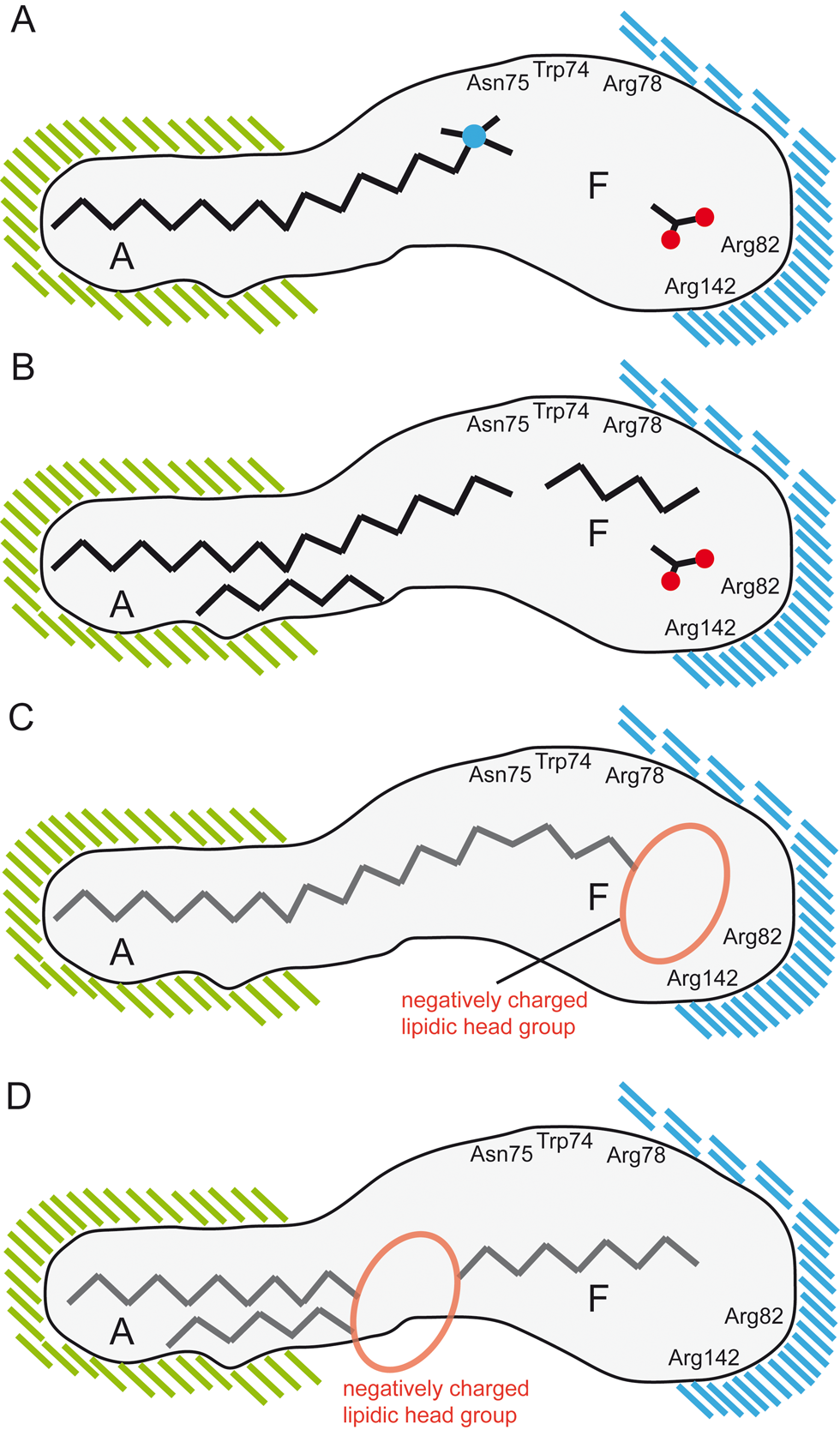

Supplement: Figure S4 — Schematic representation of YF1*7.1 ligand binding modes. The area around the A pocket is marked in green, that around the F pocket in blue; the lengths of the ligands are approximations. (A) The binding of a hydrophobic ligand with a long aliphatic chain within the A pocket is depicted (compare Figure 6A); an acetate molecule forms salt bridges with Arg82 and Arg142. (B) A hydrophobic ligand within the A pocket is shown, together with two short aliphatic molecules (compare Figure 6B); an acetate molecule compensates the charges of Arg82 and Arg142. (C) A large hydrophobic ligand with a negatively charged head group occupies most of the binding groove and interacts with the positively charged amino acids in the vicinity of the F pocket. (D) A ligand with branched hydrophobic segments rests within the binding groove; its exposed head group might interact with positively charged residues at the surface of the YF1*7.1 complex. (1.07 MB TIF) [file pbio.1000557.s004.tif]
