# Supplementary material for: Structure of a Classical MHC Class I Molecule That Binds “Non-Classical” Ligands
Source: PLoS Biol. 2010 Dec 7;8(12):e1000557. doi: 10.1371/journal.pbio.1000557 (PMC2998441; doi:10.1371/journal.pbio.1000557)
Supplement: Table S1 — Comparison of binding groove residues of YF1*7.1 as well as selected classical and non-classical class I molecules. (0.03 MB DOC) [file pbio.1000557.s005.doc]

|  | **YF1*7.1** | **BF2*2101** | **HLA-B*2709** | **H-2K^b^** | **RT1-A** | **ZAG** | **EPCR** | **ggCD1-1** | **ggCD1-2** | **hsCD1b** | **btCD1b3** | **mmCD1d** |
| --- | --- | --- | --- | --- | --- | --- | --- | --- | --- | --- | --- | --- |
| **PDB entries** | 3P37 | 3BEV | 1OF2 | 1S7Q | 1KJM | 1ZAG | 1LQV | 3JVG | 3DBX | 2H26 | 3L9R | 2FIK |
| **Total** | 27* | 32 | 33 | 29 | 32 | 33 | 43 | 38 | 23 | 49 | 47 | 46 |
| **Hydrophobic** | 16 | 16 | 9 | 8 | 11 | 16 | 31 | 34 | 19 | 40 | 34 | 34 |
| **Polar** | 7 | 9 | 13 | 13 | 15 | 11 | 9 | 4 | 4 | 6 | 8 | 8 |
| **Basic** | 2 | 5 | 6 | 4 | 3 | 3 | 2 | 0 | 0 | 1 | 3 | 2 |
| **Acidic** | 2 | 2 | 5 | 4 | 3 | 3 | 1 | 0 | 0 | 2 | 2 | 2 |
| * Number of amino acids contributing to the binding groove | | | | | | | | | | | | |
