# Supplementary material for: Structure of a Classical MHC Class I Molecule That Binds “Non-Classical” Ligands
Source: PLoS Biol. 2010 Dec 7;8(12):e1000557. doi: 10.1371/journal.pbio.1000557 (PMC2998441; doi:10.1371/journal.pbio.1000557)
Supplement: Table S2 — Crystallization and cryo-protectant conditions. (0.03 MB DOC) [file pbio.1000557.s006.doc]

| **Designation** | **Ligand*** | **Resolution** | **Crystallization** | **Cryoprotectant** |
| --- | --- | --- | --- | --- |
| L1 | - | 1.32 | 0.20 M AmAc, 0.1 M NaAc pH 5.0, 20% PEG 4000 | glycerol |
| L2 | - | 1.60 | 0.24 M AmAc, 0.1 M NaAc pH 5.0, 20% PEG 4000 | PEG 200 |
| L3 | PC | 1.65 | 0.25 M AmAc, 0.1 M NaAc pH 5.0, 19% PEG 4000 | glycerol |
| L4 | PLM | 1.55 | 0.32 M AmAc, 0.1 M NaAc pH 5.0, 16% PEG 4000 | glycerol |
| L5 | PLM | 2.60 | 0.28 M AmAc, 0.1 M NaAc pH 5.0, 14% PEG 4000 | PEG 200 |
| L6 | POPC | 1.55 | 0.25 M AmAc, 0.1 M NaAc pH 5.9, 19% PEG 4000 | PEG 200 |
| L7 | DOPC | 1.55 | 0.25 M AmAc, 0.1 M NaAc pH 5.9, 19% PEG 4000 | PEG 200 |
| L8 | OLA | 1.60 | 0.23 M AmAc, 0.1 M NaAc pH 5.0, 19% PEG 4000 | PEG 200 |
| *Ligand present in the reconstitution | | | | |
